# Structure of a dinuclear cadmium complex with 2,2′-bi­pyridine, monodentate nitrate and 3-carb­oxy-6-methyl­pyridine-2-carboxyl­ate ligands: intra­molecular carbon­yl(lone pair)⋯π(ring) and nitrate(π)⋯π(ring) inter­actions

**DOI:** 10.1107/S2056989015012384

**Published:** 2015-07-08

**Authors:** Juan Granifo, Sebastián Suarez, Ricardo Baggio

**Affiliations:** a Departamento de Ciencias Químicas y Recursos Naturales, Facultad de Ingeniería y Ciencias, Universidad de La Frontera, Casilla 54-D, Temuco, Chile; b Departamento de Química Inorgánica, Analítica y Química Física, INQUIMAE-CONICET, Facultad de Ciencias Exactas y Naturales, Universidad de Buenos Aires, Buenos Aires, Argentina; c Gerencia de Investigación y Aplicaciones, Centro Atómico Constituyentes, Comisión Nacional de Energía Atómica, Buenos Aires, Argentina

**Keywords:** crystal structure, dinuclear Cd complex, intra­molecular C—O⋯π(ring) and N—O⋯π(ring) inter­actions

## Abstract

The centrosymmetric dinuclear complex bis­(μ-3-carb­oxy-6-methyl­pyridine-2-carboxyl­ato)-κ^3^ *N*,*O* ^2^:*O* ^2^;κ^3^ *O* ^2^:*N*,*O* ^2^-bis­[(2,2′-bi­pyridine-κ^2^ *N*,*N*′)(nitrato-κ*O*)cadmium] methanol monosolvate, [Cd_2_(C_8_H_6_NO_4_)_2_(NO_3_)_2_(C_10_H_8_N_2_)_2_]·CH_3_OH, was isolated as colourless crystals from the reaction of Cd(NO_3_)_2_·4H_2_O, 6-methyl­pyridine-2,3-di­carb­oxy­lic acid (mepydcH_2_) and 2,2′-bi­pyridine in methanol. The asymmetric unit consists of a Cd^II^ cation bound to a μ-κ^3^ *N*,*O* ^2^:*O* ^2^-mepydcH^−^ anion, an *N*,*N*′-bidentate 2,2′-bi­pyridine group and an *O*-mono­dentate nitrate anion, and is completed with a methanol solvent mol­ecule at half-occupancy. The Cd complex unit is linked to its centrosymmetric image through a bridging mepydcH^−^ carboxyl­ate O atom to complete the dinuclear complex mol­ecule. Despite a significant variation in the coordination angles, indicating a considerable departure from octa­hedral coordination geometry about the Cd^II^ atom, the Cd—O and Cd—N distances in this complex are surprisingly similar. The crystal structure consists of O—H⋯O hydrogen-bonded chains parallel to *a*, further bound by C—H⋯O contacts along *b* to form planar two-dimensional arrays parallel to (001). The juxtaposed planes form inter­stitial columnar voids that are filled by the methanol solvent mol­ecules. These in turn inter­act with the complex mol­ecules to further stabilize the structure. A search in the literature showed that complexes with the mepydcH^−^ ligand are rare and complexes reported previously with this ligand do not adopt the μ-κ^3^ coordination mode found in the title compound.

## Abstract 2

A dinuclear Cd 3-carb­oxy-6-methyl­pyridine-2-carboxyl­ate complex is reported, in which the anionic ligand displays an unusual μ_2_-κ^3^ coordination mode. The crystal structure consists of hydrogen-bonded planar arrays held by *X*—H⋯O (*X* = O,*C*) and *X*—O⋯π(ring) (*X* = N,*O*) inter­actions, leaving inter­stitial columnar voids.

## Chemical context   

Pyridinedi­carboxyl­ate ligands derived from pyridine-2,3-di­carb­oxy­lic acid (*pydcH_2_*) have been extensively used in the construction of a large variety of structural motifs. The two deprotonated forms *pydcH* ^−^ and *pydc^2−^* have been shown to adopt a wide range of coordination modes through their carboxyl­ate oxygen and pyridyl nitro­gen atoms (Wang *et al.*, 2009 ▸). A search in the CSD (Version 5.3; Groom & Allen, 2014 ▸) disclosed *ca* 200 complexes displaying diverse topologies, *viz*. monomers (Gao *et al.*, 2010 ▸; Drew *et al.*, 1971 ▸), dimers (Shankar *et al.*, 2013 ▸), oligomers (Yu *et al.*, 2003 ▸) as well as one-dimensional (Semerci *et al.*, 2014 ▸), two-dimensional (Çolak *et al.*, 2011 ▸) and three-dimensional (Kanoo *et al.*, 2012 ▸) polymers. In the vast majority of cases the ligand adopts an *N,O*-chelating mode, although there are a few exceptions to this where the binding sites attach to different metal atoms (*e.g.* Wang *et al.*, 2014 ▸). By contrast, when complexes containing similar ligands but with methyl substituents in the 6-position were sought, namely those generated from 6-methyl­pyridine-2,3-di­carb­oxy­lic acid (*mepydcH*_2_), only a single structure was found involving the monoanionic *mepydcH* ^−^ ligand similar to that reported here (Gurunatha & Maji, 2009 ▸). This unique structural motif appears in the form of three isostructural, monomeric *M* ^II^ (*M* = Fe, Co, Ni) complexes [M(*bpee*)_2_(*mepydcH*)_2_] (*bpee* = 1,2-bis­(4-pyrid­yl)ethyl­ene) with octa­hedral geometry around *M* ^II^. Both *mepydcH* ^−^ fragments act in a simple κ^2^ *N,O^2^*-chelating mode binding to a single nucleus while the two N-bound *bpee* ligands are *trans*-monodentate. The formation of these mononuclear complexes is unusual considering the obvious bridging potential of the *bpee* ligands. Mixed-ligand complexes based on non-methyl­ated 2,3-pyridinedi­carboxyl­ate and 4,4′-bi­pyridine-like ligands usually generate stable polymeric structures with the *exo*-bidentate ligands adopting a bridging role (Kanoo *et al.*, 2012 ▸; Wang *et al.*, 2009 ▸; Maji *et al.*, 2005 ▸).
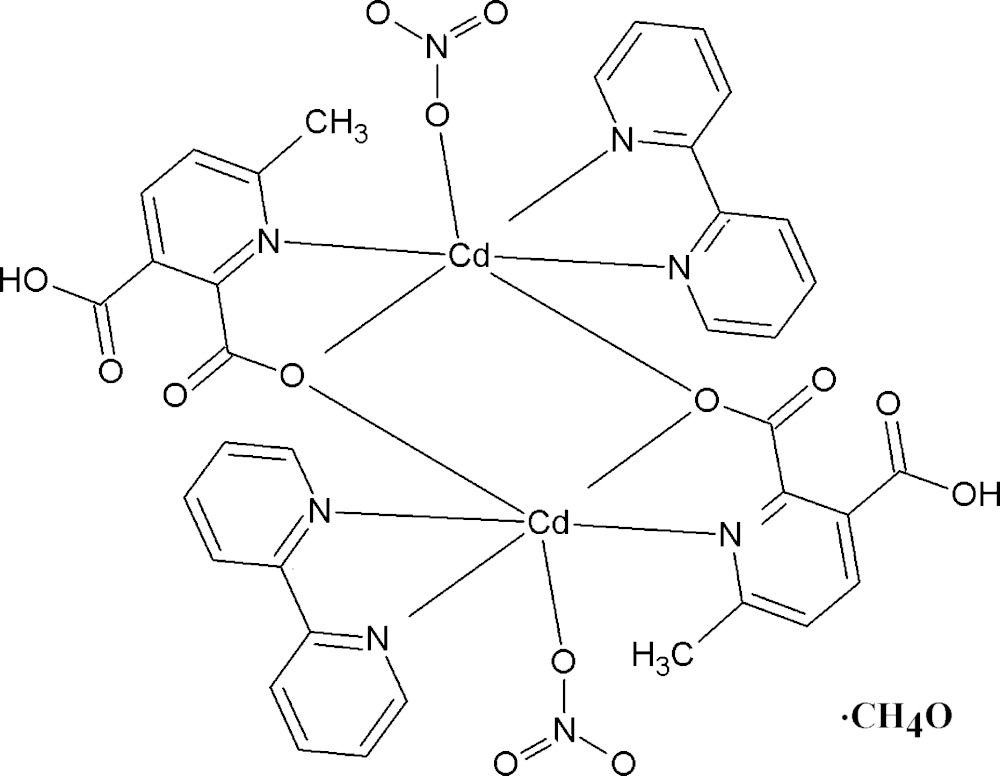


In an attempt to understand the coordination behaviour of this unusual monoanionic *mepydcH* ^−^ ligand better, we report the structure of the dinuclear complex [Cd_2_(2,2′-bi­pyridine)_2_(*mepydcH*)_2_(NO_3_)_2_]·MeOH (I). The uncommon bridging-chelating μ_2_-(κ^3^ *N,O^2^*:*O^2^*) coordination behaviour and the fact that the ligand is only singly deprotonated has no counterpart in complexes of the non-methyl­ated ligands and makes this a genuinely novel structure. The closest relatives with 2,2′-bi­pyridine as the auxiliary ligand are found with di-anionic *pydc^2−^* ligands, but these are either mononuclear (Wang & Okabe, 2005 ▸) or form coordination polymers (Li *et al.*, 2013 ▸; Yin & Liu, 2009 ▸; Zhang *et al.* 2013 ▸).

## Structural commentary   

**Figure 1:**
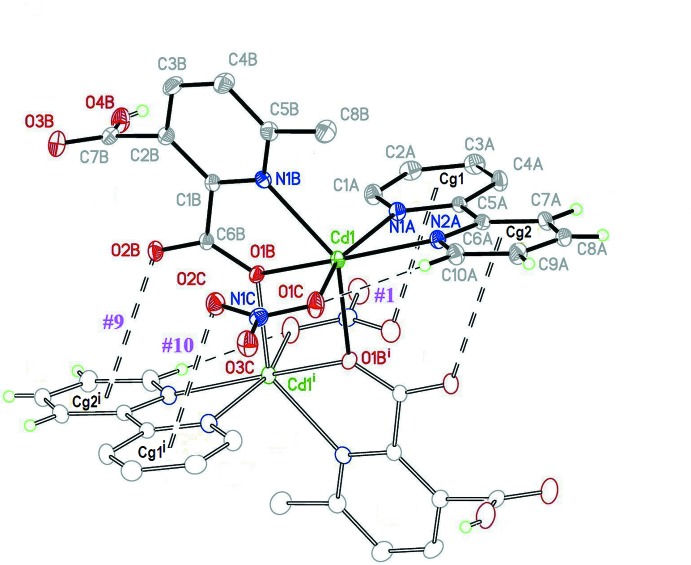
Displacement ellipsoid plot of (I) (with 40% probability ellipsoids), showing the dimeric unit with atom and ring labelling. Inter­actions within the dimeric unit are also shown, C—H⋯O as dashed lines, C—O⋯π(ring) as double-dashed lines. For symmetry codes see Tables 2 ▸ and 3 ▸; additional symmetry code: (i) 1 − *x*, 1 − *y*, 1 − *z*.

The complex consists of a Cd^II^ cation to which a singly protonated 3-carb­oxy-6-methyl­pyridine-2-carboxyl­ate ion (*mepydcH* ^−^) chelates through the pyridine N and carboxyl­ate O atoms. A chelating 2,2′-bi­pyridine that binds through both nitro­gen atoms and a unidentate nitrate anion complete the coordination sphere; the asymmetric unit also contains a non-coordinating half-occupancy methanol solvate. This five coordinate Cd^II^ unit, in turn, binds to its centrosymmetric image through the carboxyl­ate oxygen atom of the *mepydcH* ^−^ ligand, forming a pair of Cd–O–Cd bridges. As a result, a dimeric unit forms (Fig. 1 ▸) with each Cd^II^ atom in a six-coordinate N_3_O_3_ ligand environment. The Cd—*X* (*X* = N or O) distances are reasonable, spanning the range 2.304 (2)–2.332 (3) Å. However, the coordination angles vary widely [*X*–Cd–*X* ranges: *cis* 71.15 (10)–115.79 (9)°; *trans* 142.36 (8)–159.48 (9)°]; the result is a rather distorted octa­hedral geometry around Cd1. Selected geometric parameters are shown in Table 1 ▸; the bridging Cd—O distances are the shortest in the coordination sphere, 2.304 (2) and 2.310 (2) Å, resulting in a Cd⋯Cd separation of 3.700 (3) Å. This value is slightly larger than the mean for similar environments found in the CSD (3.61 Å for 885 cases), though well within the sample standard deviation (0.22 Å).

## Supra­molecular features   

The crystal structure, made up of isolated dimers, is sustained by three different types of non-covalent inter­action, *viz*., hydrogen bonds (Table 2 ▸), C=O⋯π and nitrate(π)⋯π contacts (Table 3 ▸). These inter­actions can be clearly differentiated according to the substructure that they support:

**a**) Contacts #1 (Table 2 ▸) and #9, #10 (Table 3 ▸) are inter­nal to the dinuclear motif, as shown in Fig. 1 ▸. The first one links the bi­pyridine C10*A*—H10*A* group with the coordinating nitrate oxygen O1*C*. Contact #9 is a typical lone pair–π inter­action with a dihedral angle of 72.19° between the carboxyl­ate and the ring plane, and a C—O⋯*Cg*2 angle of 126.63°. These values are close to those for the ideal geometry (90° and 120°, respectively) when a lone pair provided by a carbonyl oxygen points toward the centroid of an aromatic ring (Egli & Sarkhel, 2007 ▸). By contrast, in contact #10 the orientation of the nitrate plane is more or less parallel to the ring plane (6.84°), suggesting a π–π inter­action with the π-orbitals of the nitrate fragment inter­acting with those of the aromatic ring. A similar argument has already been applied by Frontera *et al.* (2011 ▸) and García-Raso *et al.* (2009 ▸) when nitrate anions inter­act with pyrimidinium rings. These carbon­yl(lone pair)⋯π(ring) (#9) and nitrate(π)⋯π(ring) (#10) inter­actions in (I) fulfill a relevant function, serving to strengthen the dimeric unit (Fig. 1 ▸).

**Figure 2:**
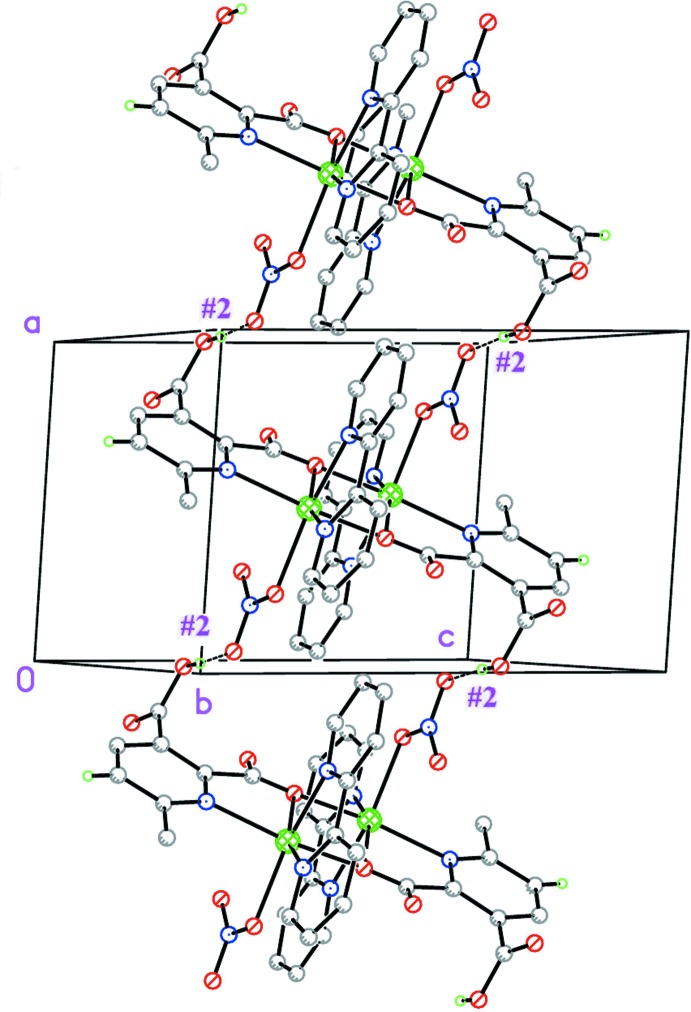
The [100] chain defined by O—H⋯O inter­action #2 (Table 2 ▸).

**b**) Strong inter­molecular O—H⋯O contacts #2 (Table 2 ▸) involving the hydrogen atom of the free carb­oxy­lic acid group of the *mepydcH* ^−^ ligand with a non-bonded oxygen atom of a nitrate ligand, has the pivotal action of linking the dimers along *a*, forming chains parallel to [100] (Fig. 2 ▸).

**Figure 3:**
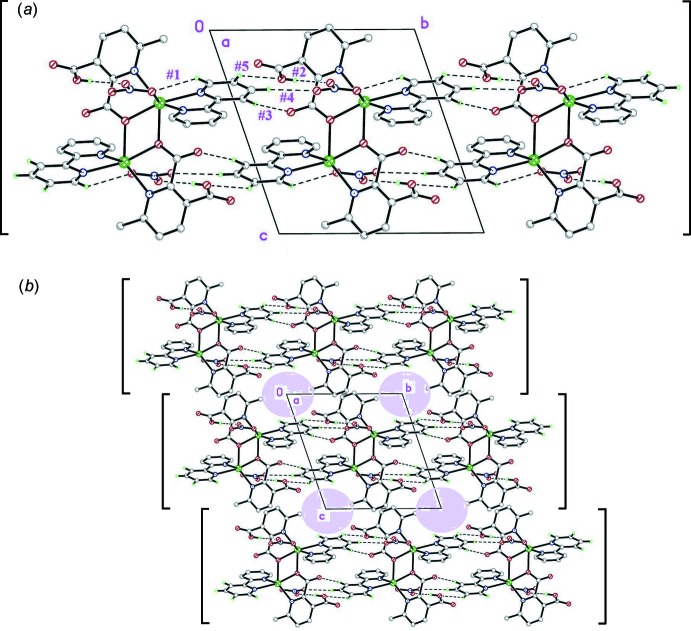
Two projections along [100], presenting (within square brakets) views of the two-dimensional substructures parallel to (001), formed by the [100] columns linked along *b*. (*a*) Showing a single plane, with inter­action details. (*b*) Displaying the columnar voids (coloured) generated by juxtaposition of the planes.

**c**) C—H⋯O inter­actions #3, #4 and #5 (Table 2 ▸), in turn, serve to link the above chains laterally along *b*, to form 2D substructures parallel to (001) (Fig. 3 ▸ *a*). These planes juxtapose along [001] with rather weak direct inter­actions. In the process, however, significant columnar voids parallel to the chains are formed (with a volume 13% of the total cell volume, Fig. 3 ▸ *b*) in which the partial occupancy methanol solvate mol­ecules reside. These are not free, but enter instead into a number of weak C—H⋯O, O—H⋯O and C—H⋯π interactions (#6, #7 and #8 in Table 2 ▸) linking them to a framework of complex mol­ecules, further stabilizing the structure.

## ATR (attenuated total reflectance) FT–IR spectroscopy   

**Figure 4:**
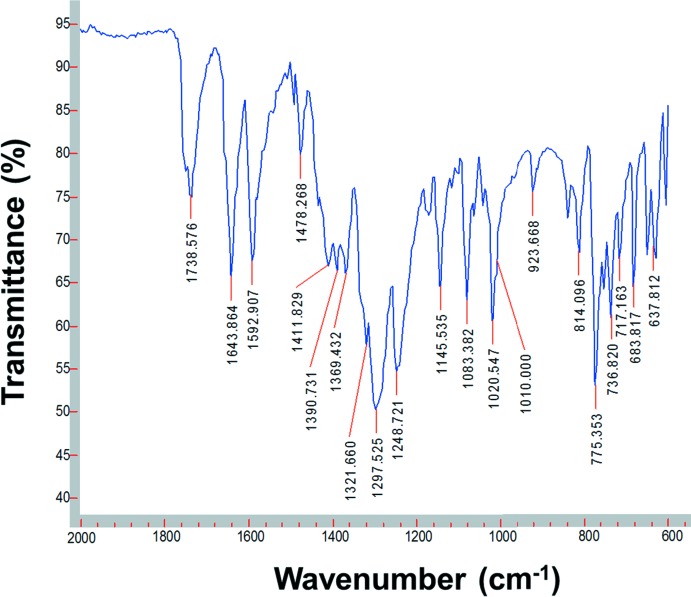
FT–IR spectrum of (I)

The IR spectra of *mepydcH*_2_, 2.2′-bi­pyridine and (I) were recorded on an Agilent Cary 630 FT–IR spectrometer with Varian *Resolutions Pro* software, using a Diamond ATR accessory. The FT–IR spectrum of (I) (Fig. 4 ▸) was recorded in the 4000–600 cm^−1^ range, and confirms the structural data indicating the presence of the coordinating nitrate and *mepydcH* ^−^ anions. Bands due to the unidentate NO_3_^−^ group were found at 1478 and 1298 cm^−1^ and appear due to the ν_asym_(ONO) and ν_sym_(ONO) vibrations, with a shoulder at 1010 cm^−1^ due to the ν(NO) stretching modes of nitrate groups (Nakamoto, 1997 ▸). The carb­oxy­lic acid group (COOH) of the *mepydcH* ^−^ ligand in complex (I) is identified by a weak band at 3083 cm^−1^, ν(OH) stretching for a hydrogen-bonded system (Alisir *et al.*, 2013 ▸), and a very strong band at 1738 cm^−1^, ν(C=O) stretch. The deprotonated carboxyl­ate (COO^−^) is characterized by the asymmetric and symmetric stretching modes ν_as_ at 1593 cm^−1^ and ν_s_ at 1322 cm^−1^. This confirms the unidentate coordination of the carboxyl­ate O atom, with the difference between these frequencies being > 200 cm^−1^(Δ = ν_as_ − ν_s_ = 271 cm^−1^) (Deacon & Phillips, 1980 ▸). Finally, around 1400 cm^−1^, a set of three bands appears (1412, 1391 and 1369 cm^−1^) of almost equal intensity due to the ν(C=C) + ν(C=N) vibrations from the coordinating 2,2′-bi­pyridine ligand (Yan *et al.*, 2011 ▸).

## Synthesis and crystallization   

Solid 2,2′-bi­pyridine (0.031 g, 0.20 mmol) was added to a solution prepared by disolving Cd(NO_3_)·4H_2_O (0.062 g, 0.20 mmol) and *mepydcH*_2_ (0.036 g, 0.20 mmol) in MeOH (4.0 mL). The mixture was stirred to dissolve the 2,2′-bi­pyridine and was then allowed to stand undisturbed at room temperature in an uncovered 10 mL beaker. Colourless single crystals of compound (I) suitable for X-ray diffraction were obtained within 8 h. The crystals were separated by filtration, washed with MeOH (2 x 2 mL) and diethyl ether (2 x 3 mL) (yield: 0.045 g, 44%).

## Refinement   

Relevant crystallographic data for (I) as well as pertinent experimental details are provided in Table 4 ▸. H atoms bonded to C were found in a difference Fourier map, but were then idealized and refined as riding atoms; C—H_arom_: 0.93 Å, *U*_eq_(H) = 1.2*U*_eq_(C); C—H_meth­yl_: 0.97 Å, *U*_eq_(H) = 1.5*U*_eq_(C). The O—H hydrogen atom was refined with a restrained O—H distance [0.85 (1)Å], and with *U*(H) = 1.2*U*_eq_(O). The methanol solvate was refined at half occupancy.

## Supplementary Material

Crystal structure: contains datablock(s) I, global. DOI: 10.1107/S2056989015012384/sj5468sup1.cif

Structure factors: contains datablock(s) I. DOI: 10.1107/S2056989015012384/sj5468Isup2.hkl

CCDC reference: 1409269

Additional supporting information:  crystallographic information; 3D view; checkCIF report

## Figures and Tables

**Table 1:** Selected bond lengths ()

Cd1O1*B* ^i^	2.304(2)	Cd1N1*A*	2.323(3)
Cd1O1*B*	2.310(2)	Cd1O1*C*	2.329(2)
Cd1N2*A*	2.310(3)	Cd1N1*B*	2.332(3)

Symmetry code: (i) 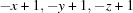.

**Table 2:** Hydrogen-bonding interactions (, ) in (I) *Cg*1 is the centroid of the N1*A*/C1*A*C5*A* ring.

Int.#	DHA	DH	HA	DA	DHA
#1	C10*A*H10*A*O1*C*	0.93	2.52	3.143(5)	124
#2	O4*B*H4*BO*O3*C* ^ii^	0.84(3)	1.83(3)	2.670(5)	176(6)
#3	C7*A*H7*A*O2*B* ^iii^	0.93	2.42	3.339(4)	168
#4	C8*A*H8*A*O2*C* ^iii^	0.93	2.59	3.51(4)	167
#5	C9*A*H9*A*O4*B* ^iv^	0.93	2.53	3.186(5)	127
#6	C8*B*H8*BC*O1*M*	0.960	2.54	3.361(8)	144
#7	O1*M*H1*M*O3*B* ^iii^	0.85(5)	2.42(9)	2.951(9)	121(9)
#8	C1*M*H1*M*3*Cg*1	0.96	2.78	3.640	149

Symmetry codes: (ii) 1+*x*, *y*, *z*; (iii) *x*, 1+*y*, *z*; (iv) 1+*x*, 1+*y*, *z*.

**Table 3:** *X*O interactions (, )in (I) *Cg*1 is the centroid of the N1*A*/C1*A*C5*A* ring and *Cg*2 is the centroid of the N2*A*/C6*A*C10*A* ring.

Int.#	*X*O*Cg*	O*Cg*	*X*O*Cg*
#9	C6*B*O2*B* *Cg*2^i^	3.637(3)	126.6(2)
#10	N1*C*O2*C* *Cg*1^i^	3.442(4)	104.2(2)

Symmetry code: (i) 1*x*, 1*y*, 1*z*.

**Table 4:** Experimental details

Crystal data	
Chemical formula	[Cd_2_(C_8_H_6_NO_4_)_2_(NO_3_)_2_(C_10_H_8_N_2_)_2_]CH_4_O
*M*_r_	1053.50
Crystal system, space group	Triclinic, *P* 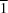
Temperature (K)	295
*a*, *b*, *c* ()	8.4096(5), 10.9626(6), 11.5056(4)
, , ()	71.241(4), 86.537(4), 86.803(5)
*V* (^3^)	1001.79(9)
*Z*	1
Radiation type	Mo *K*
(mm^1^)	1.14
Crystal size (mm)	0.36 0.14 0.10

Data collection	
Diffractometer	Oxford Diffraction Gemini CCD S Ultra
Absorption correction	Multi-scan (*CrysAlis PRO*; Oxford Diffraction, 2009 ▸)
No. of measured, independent and observed [*I* > 2(*I*)] reflections	21744, 4819, 4155
*R*_int_	0.057
(sin /)_max_ (^1^)	0.684

Refinement	
*R*[*F* ^2^ > 2(*F* ^2^)], *wR*(*F* ^2^), *S*	0.036, 0.092, 1.01
No. of reflections	4819
No. of parameters	298
No. of restraints	4
H-atom treatment	H atoms treated by a mixture of independent and constrained refinement
_max_, _min_ (e ^3^)	1.07, 0.74

Computer programs: *CrysAlis PRO* (Oxford Diffraction, 2009 ▸), *SHELXS97* and *SHELXTL* (Sheldrick, 2008 ▸), *SHELXL2014* (Sheldrick, 2015 ▸) and *PLATON* (Spek, 2009 ▸).

## supplementary crystallographic information

### Crystal data

**Table:**

[Cd_2_(C_8_H_6_NO_4_)_2_(NO_3_)_2_(C_10_H_8_N_2_)_2_]·CH_4_O	*Z* = 1
*M**_r_* = 1053.50	*F*(000) = 526
Triclinic, *P*1	*D*_x_ = 1.746 Mg m^−^^3^
*a* = 8.4096 (5) Å	Mo *K*α radiation, λ = 0.71073 Å
*b* = 10.9626 (6) Å	Cell parameters from 2675 reflections
*c* = 11.5056 (4) Å	θ = 3.8–28.8°
α = 71.241 (4)°	µ = 1.14 mm^−^^1^
β = 86.537 (4)°	*T* = 295 K
γ = 86.803 (5)°	Block, colourless
*V* = 1001.79 (9) Å^3^	0.36 × 0.14 × 0.10 mm

### Data collection

**Table:**

Oxford Diffraction Gemini CCD S Ultra diffractometer	4155 reflections with *I* > 2σ(*I*)
Radiation source: fine-focus sealed tube	*R*_int_ = 0.057
ω scans, thick slices	θ_max_ = 29.1°, θ_min_ = 3.6°
Absorption correction: multi-scan (*CrysAlis PRO*; Oxford Diffraction, 2009)	*h* = −11→11
*T*_min_ = ?, *T*_max_ = ?	*k* = −14→14
21744 measured reflections	*l* = −15→15
4819 independent reflections	

### Refinement

**Table:**

Refinement on *F*^2^	4 restraints
Least-squares matrix: full	Hydrogen site location: mixed
*R*[*F*^2^ > 2σ(*F*^2^)] = 0.036	H atoms treated by a mixture of independent and constrained refinement
*wR*(*F*^2^) = 0.092	*w* = 1/[σ^2^(*F*_o_^2^) + (0.0394*P*)^2^ + 1.5425*P*] where *P* = (*F*_o_^2^ + 2*F*_c_^2^)/3
*S* = 1.01	(Δ/σ)_max_ < 0.001
4819 reflections	Δρ_max_ = 1.07 e Å^−^^3^
298 parameters	Δρ_min_ = −0.74 e Å^−^^3^

### Special details

**Table:**

Geometry. All e.s.d.'s (except the e.s.d. in the dihedral angle between two l.s. planes) are estimated using the full covariance matrix. The cell e.s.d.'s are taken into account individually in the estimation of e.s.d.'s in distances, angles and torsion angles; correlations between e.s.d.'s in cell parameters are only used when they are defined by crystal symmetry. An approximate (isotropic) treatment of cell e.s.d.'s is used for estimating e.s.d.'s involving l.s. planes.

### Fractional atomic coordinates and isotropic or equivalent isotropic displacement parameters (Å^2^)

**Table:**

	*x*	*y*	*z*	*U*_iso_*/*U*_eq_	Occ. (<1)
Cd1	0.47993 (3)	0.63548 (2)	0.35527 (2)	0.01961 (9)	
N1A	0.6981 (3)	0.7364 (3)	0.3922 (3)	0.0229 (6)	
N2A	0.4198 (3)	0.8540 (3)	0.3047 (2)	0.0223 (6)	
C1A	0.8378 (4)	0.6740 (4)	0.4272 (3)	0.0279 (7)	
H1A	0.8516	0.5882	0.4306	0.033*	
C2A	0.9614 (4)	0.7331 (4)	0.4581 (3)	0.0292 (8)	
H2A	1.0575	0.6883	0.4821	0.035*	
C3A	0.9393 (4)	0.8601 (4)	0.4526 (3)	0.0312 (8)	
H3A	1.0204	0.9020	0.4740	0.037*	
C4A	0.7968 (4)	0.9252 (4)	0.4152 (3)	0.0286 (7)	
H4A	0.7812	1.0114	0.4104	0.034*	
C5A	0.6771 (4)	0.8603 (3)	0.3851 (3)	0.0196 (6)	
C6A	0.5211 (4)	0.9244 (3)	0.3409 (3)	0.0201 (6)	
C7A	0.4801 (4)	1.0501 (3)	0.3369 (3)	0.0263 (7)	
H7A	0.5504	1.0976	0.3629	0.032*	
C8A	0.3343 (4)	1.1041 (3)	0.2940 (3)	0.0286 (8)	
H8A	0.3048	1.1877	0.2920	0.034*	
C9A	0.2327 (4)	1.0328 (3)	0.2540 (3)	0.0303 (8)	
H9A	0.1348	1.0679	0.2230	0.036*	
C10A	0.2801 (4)	0.9084 (3)	0.2614 (3)	0.0280 (7)	
H10A	0.2116	0.8599	0.2351	0.034*	
O1B	0.6097 (3)	0.4384 (2)	0.44192 (19)	0.0216 (5)	
O2B	0.7028 (3)	0.2598 (2)	0.4031 (2)	0.0256 (5)	
O3B	0.8147 (3)	0.1816 (3)	0.1644 (3)	0.0390 (6)	
O4B	0.9923 (3)	0.2717 (3)	0.2400 (3)	0.0340 (6)	
H4BO	1.004 (6)	0.344 (2)	0.248 (4)	0.051 (14)*	
N1B	0.5964 (3)	0.5661 (3)	0.1972 (2)	0.0200 (5)	
C1B	0.6745 (4)	0.4503 (3)	0.2349 (3)	0.0185 (6)	
C2B	0.7605 (4)	0.3994 (3)	0.1523 (3)	0.0226 (7)	
C3B	0.7607 (5)	0.4714 (4)	0.0288 (3)	0.0316 (8)	
H3B	0.8164	0.4402	−0.0288	0.038*	
C4B	0.6796 (5)	0.5880 (4)	−0.0090 (3)	0.0343 (9)	
H4B	0.6799	0.6361	−0.0919	0.041*	
C5B	0.5963 (4)	0.6345 (3)	0.0780 (3)	0.0255 (7)	
C6B	0.6608 (4)	0.3736 (3)	0.3714 (3)	0.0185 (6)	
C7B	0.8543 (4)	0.2739 (3)	0.1893 (3)	0.0265 (7)	
C8B	0.5036 (5)	0.7606 (4)	0.0418 (3)	0.0376 (9)	
H8BA	0.5131	0.7999	−0.0457	0.056*	
H8BB	0.3934	0.7462	0.0669	0.056*	
H8BC	0.5448	0.8166	0.0811	0.056*	
N1C	0.1872 (3)	0.5118 (3)	0.2872 (2)	0.0248 (6)	
O1C	0.2228 (3)	0.6168 (3)	0.3000 (3)	0.0350 (6)	
O2C	0.2871 (3)	0.4227 (3)	0.2993 (3)	0.0341 (6)	
O3C	0.0460 (3)	0.5009 (3)	0.2619 (3)	0.0374 (6)	
O1M	0.7710 (10)	0.9624 (9)	0.0748 (8)	0.072 (2)	0.5
H1M	0.816 (4)	1.0346 (13)	0.046 (17)	0.18 (9)*	0.5
C1M	0.8993 (11)	0.8676 (10)	0.0932 (8)	0.060 (3)	0.5
H1M1	0.9936	0.9068	0.0490	0.090*	0.5
H1M2	0.8713	0.7994	0.0637	0.090*	0.5
H1M3	0.9189	0.8331	0.1792	0.090*	0.5

### Atomic displacement parameters (Å^2^)

**Table:**

	*U*^11^	*U*^22^	*U*^33^	*U*^12^	*U*^13^	*U*^23^
Cd1	0.02114 (13)	0.01596 (13)	0.02201 (13)	0.00148 (9)	−0.00289 (9)	−0.00650 (9)
N1A	0.0207 (14)	0.0214 (14)	0.0274 (14)	−0.0001 (11)	−0.0030 (11)	−0.0087 (11)
N2A	0.0226 (14)	0.0178 (14)	0.0259 (14)	0.0016 (11)	−0.0031 (11)	−0.0062 (11)
C1A	0.0255 (17)	0.0252 (18)	0.0331 (18)	0.0027 (14)	−0.0037 (14)	−0.0095 (15)
C2A	0.0201 (16)	0.038 (2)	0.0293 (18)	−0.0008 (15)	−0.0038 (14)	−0.0105 (16)
C3A	0.0261 (18)	0.040 (2)	0.0324 (19)	−0.0088 (16)	−0.0041 (15)	−0.0166 (16)
C4A	0.0336 (19)	0.0228 (18)	0.0327 (18)	−0.0046 (15)	−0.0020 (15)	−0.0129 (15)
C5A	0.0216 (15)	0.0188 (16)	0.0192 (14)	−0.0028 (12)	0.0002 (12)	−0.0069 (12)
C6A	0.0231 (16)	0.0182 (16)	0.0181 (14)	−0.0022 (13)	0.0004 (12)	−0.0048 (12)
C7A	0.0312 (18)	0.0193 (17)	0.0279 (17)	−0.0039 (14)	0.0003 (14)	−0.0069 (14)
C8A	0.036 (2)	0.0169 (17)	0.0293 (18)	0.0031 (14)	0.0055 (15)	−0.0043 (14)
C9A	0.0277 (18)	0.0239 (18)	0.0374 (19)	0.0089 (15)	−0.0044 (15)	−0.0083 (15)
C10A	0.0248 (17)	0.0228 (18)	0.0363 (19)	0.0030 (14)	−0.0067 (14)	−0.0089 (15)
O1B	0.0278 (12)	0.0180 (11)	0.0185 (11)	0.0049 (9)	0.0004 (9)	−0.0062 (9)
O2B	0.0328 (13)	0.0168 (12)	0.0271 (12)	0.0025 (10)	−0.0033 (10)	−0.0073 (10)
O3B	0.0471 (17)	0.0307 (15)	0.0461 (16)	0.0020 (12)	−0.0037 (13)	−0.0221 (13)
O4B	0.0265 (13)	0.0314 (15)	0.0481 (16)	0.0076 (11)	−0.0078 (11)	−0.0182 (13)
N1B	0.0196 (13)	0.0185 (14)	0.0216 (13)	−0.0010 (11)	−0.0033 (10)	−0.0055 (11)
C1B	0.0173 (14)	0.0193 (16)	0.0208 (15)	−0.0016 (12)	−0.0047 (12)	−0.0082 (12)
C2B	0.0197 (16)	0.0245 (17)	0.0259 (16)	−0.0017 (13)	−0.0012 (13)	−0.0110 (14)
C3B	0.038 (2)	0.035 (2)	0.0234 (17)	0.0039 (16)	0.0038 (15)	−0.0126 (15)
C4B	0.047 (2)	0.032 (2)	0.0203 (16)	0.0064 (17)	−0.0004 (15)	−0.0051 (15)
C5B	0.0295 (18)	0.0226 (17)	0.0234 (16)	0.0013 (14)	−0.0052 (13)	−0.0055 (13)
C6B	0.0156 (14)	0.0181 (16)	0.0237 (15)	−0.0006 (12)	−0.0029 (12)	−0.0088 (12)
C7B	0.0265 (17)	0.0270 (19)	0.0283 (17)	0.0012 (14)	0.0046 (14)	−0.0135 (15)
C8B	0.051 (2)	0.032 (2)	0.0256 (18)	0.0103 (18)	−0.0058 (17)	−0.0053 (16)
N1C	0.0250 (15)	0.0292 (16)	0.0218 (13)	−0.0013 (12)	−0.0019 (11)	−0.0104 (12)
O1C	0.0264 (13)	0.0315 (15)	0.0561 (17)	0.0001 (11)	−0.0092 (12)	−0.0254 (13)
O2C	0.0268 (13)	0.0282 (14)	0.0484 (16)	0.0019 (11)	−0.0004 (11)	−0.0143 (12)
O3C	0.0253 (13)	0.0381 (16)	0.0555 (17)	−0.0034 (11)	−0.0108 (12)	−0.0224 (14)
O1M	0.078 (6)	0.075 (6)	0.077 (5)	−0.016 (5)	−0.010 (4)	−0.039 (5)
C1M	0.052 (6)	0.097 (9)	0.036 (5)	−0.018 (6)	−0.010 (4)	−0.023 (5)

### Geometric parameters (Å, º)

**Table:**

Cd1—O1B^i^	2.304 (2)	O1B—C6B	1.281 (4)
Cd1—O1B	2.310 (2)	O2B—C6B	1.220 (4)
Cd1—N2A	2.310 (3)	O3B—C7B	1.205 (4)
Cd1—N1A	2.323 (3)	O4B—C7B	1.326 (4)
Cd1—O1C	2.329 (2)	O4B—H4BO	0.845 (10)
Cd1—N1B	2.332 (3)	N1B—C5B	1.336 (4)
N1A—C5A	1.336 (4)	N1B—C1B	1.348 (4)
N1A—C1A	1.342 (4)	C1B—C2B	1.397 (4)
N2A—C10A	1.336 (4)	C1B—C6B	1.525 (4)
N2A—C6A	1.349 (4)	C2B—C3B	1.386 (5)
C1A—C2A	1.377 (5)	C2B—C7B	1.496 (5)
C1A—H1A	0.9300	C3B—C4B	1.367 (5)
C2A—C3A	1.375 (5)	C3B—H3B	0.9300
C2A—H2A	0.9300	C4B—C5B	1.398 (5)
C3A—C4A	1.378 (5)	C4B—H4B	0.9300
C3A—H3A	0.9300	C5B—C8B	1.497 (5)
C4A—C5A	1.387 (5)	C8B—H8BA	0.9600
C4A—H4A	0.9300	C8B—H8BB	0.9600
C5A—C6A	1.490 (4)	C8B—H8BC	0.9600
C6A—C7A	1.389 (5)	N1C—O2C	1.230 (4)
C7A—C8A	1.379 (5)	N1C—O3C	1.260 (4)
C7A—H7A	0.9300	N1C—O1C	1.261 (4)
C8A—C9A	1.380 (5)	O1M—C1M	1.431 (8)
C8A—H8A	0.9300	O1M—H1M	0.855 (10)
C9A—C10A	1.377 (5)	C1M—H1M1	0.9600
C9A—H9A	0.9300	C1M—H1M2	0.9600
C10A—H10A	0.9300	C1M—H1M3	0.9600
			
O1B^i^—Cd1—O1B	73.38 (8)	N2A—C10A—C9A	123.1 (3)
O1B^i^—Cd1—N2A	101.83 (9)	N2A—C10A—H10A	118.4
O1B—Cd1—N2A	159.48 (9)	C9A—C10A—H10A	118.4
O1B^i^—Cd1—N1A	94.90 (9)	C6B—O1B—Cd1^i^	128.9 (2)
O1B—Cd1—N1A	89.17 (9)	C6B—O1B—Cd1	118.42 (19)
N2A—Cd1—N1A	71.15 (10)	Cd1^i^—O1B—Cd1	106.62 (8)
O1B^i^—Cd1—O1C	88.36 (9)	C7B—O4B—H4BO	109 (3)
O1B—Cd1—O1C	112.97 (9)	C5B—N1B—C1B	120.4 (3)
N2A—Cd1—O1C	86.44 (9)	C5B—N1B—Cd1	124.9 (2)
N1A—Cd1—O1C	157.56 (10)	C1B—N1B—Cd1	114.7 (2)
O1B^i^—Cd1—N1B	142.36 (8)	N1B—C1B—C2B	121.7 (3)
O1B—Cd1—N1B	71.66 (8)	N1B—C1B—C6B	117.5 (3)
N2A—Cd1—N1B	115.79 (9)	C2B—C1B—C6B	120.7 (3)
N1A—Cd1—N1B	98.09 (9)	C3B—C2B—C1B	117.6 (3)
O1C—Cd1—N1B	92.65 (9)	C3B—C2B—C7B	118.4 (3)
C5A—N1A—C1A	119.8 (3)	C1B—C2B—C7B	124.1 (3)
C5A—N1A—Cd1	117.1 (2)	C4B—C3B—C2B	120.4 (3)
C1A—N1A—Cd1	123.0 (2)	C4B—C3B—H3B	119.8
C10A—N2A—C6A	118.9 (3)	C2B—C3B—H3B	119.8
C10A—N2A—Cd1	123.4 (2)	C3B—C4B—C5B	119.6 (3)
C6A—N2A—Cd1	116.7 (2)	C3B—C4B—H4B	120.2
N1A—C1A—C2A	122.1 (3)	C5B—C4B—H4B	120.2
N1A—C1A—H1A	119.0	N1B—C5B—C4B	120.4 (3)
C2A—C1A—H1A	119.0	N1B—C5B—C8B	117.7 (3)
C3A—C2A—C1A	118.3 (3)	C4B—C5B—C8B	121.9 (3)
C3A—C2A—H2A	120.9	O2B—C6B—O1B	126.7 (3)
C1A—C2A—H2A	120.9	O2B—C6B—C1B	118.0 (3)
C2A—C3A—C4A	119.9 (3)	O1B—C6B—C1B	115.3 (3)
C2A—C3A—H3A	120.0	O3B—C7B—O4B	120.6 (3)
C4A—C3A—H3A	120.0	O3B—C7B—C2B	122.0 (3)
C3A—C4A—C5A	118.9 (3)	O4B—C7B—C2B	117.1 (3)
C3A—C4A—H4A	120.5	C5B—C8B—H8BA	109.5
C5A—C4A—H4A	120.5	C5B—C8B—H8BB	109.5
N1A—C5A—C4A	121.0 (3)	H8BA—C8B—H8BB	109.5
N1A—C5A—C6A	116.7 (3)	C5B—C8B—H8BC	109.5
C4A—C5A—C6A	122.3 (3)	H8BA—C8B—H8BC	109.5
N2A—C6A—C7A	121.0 (3)	H8BB—C8B—H8BC	109.5
N2A—C6A—C5A	116.6 (3)	O2C—N1C—O3C	121.1 (3)
C7A—C6A—C5A	122.4 (3)	O2C—N1C—O1C	121.0 (3)
C8A—C7A—C6A	119.4 (3)	O3C—N1C—O1C	117.9 (3)
C8A—C7A—H7A	120.3	N1C—O1C—Cd1	118.4 (2)
C6A—C7A—H7A	120.3	C1M—O1M—H1M	104.7 (13)
C7A—C8A—C9A	119.4 (3)	O1M—C1M—H1M1	109.5
C7A—C8A—H8A	120.3	O1M—C1M—H1M2	109.5
C9A—C8A—H8A	120.3	H1M1—C1M—H1M2	109.5
C10A—C9A—C8A	118.2 (3)	O1M—C1M—H1M3	109.5
C10A—C9A—H9A	120.9	H1M1—C1M—H1M3	109.5
C8A—C9A—H9A	120.9	H1M2—C1M—H1M3	109.5

Symmetry code: (i) −*x*+1, −*y*+1, −*z*+1.
